# Supplementary material for: Splice-Junction-Based Mapping of Alternative Isoforms in the Human Proteome
Source: Cell Rep. Author manuscript; Available in PMC 2020 Jan 15. (PMC6961840; doi:10.1016/j.celrep.2019.11.026)
Supplement: 2 [file NIHMS1546469-supplement-2.zip › DF1/PXD010154/Pancreas/Pancreas_1_TRIM28_SAAAAASAASGSPGPGEGSAGGEKR.pdf]

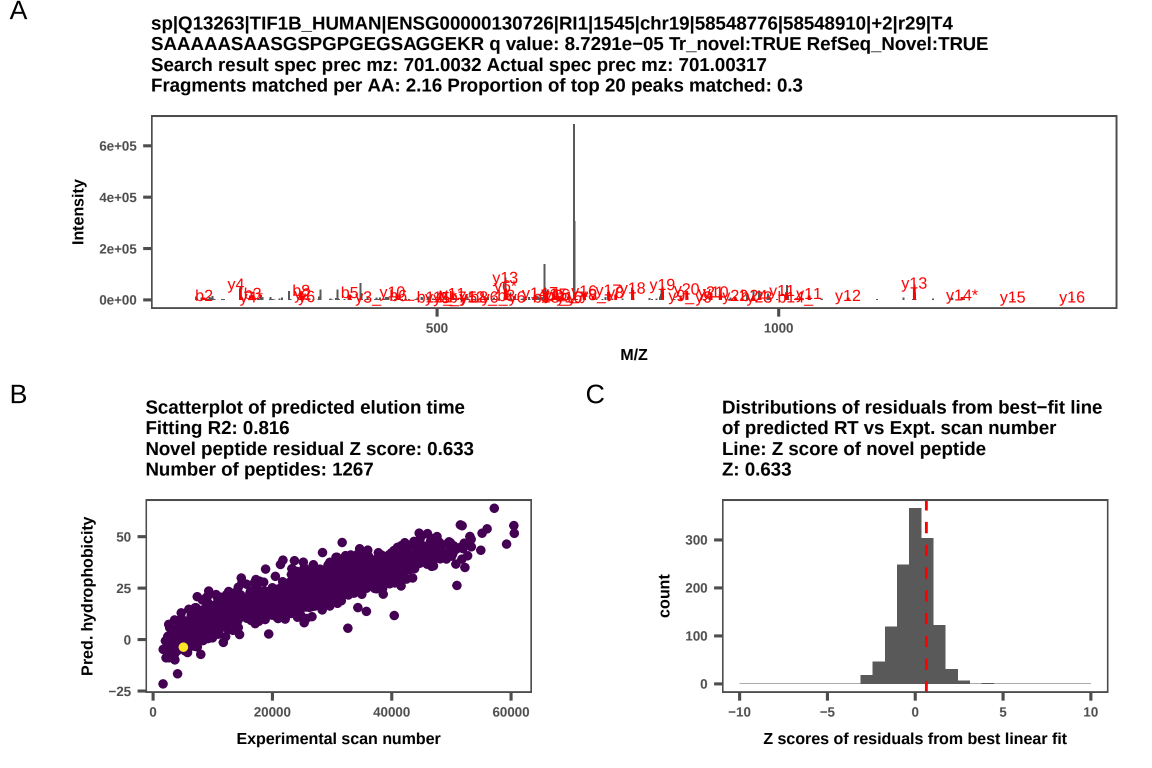
A
sp|Q13263|TIF1B_HUMAN|ENSG00000130726|RI1|1545|chr19|58548776|58548910|+2|r29|T4
SAAAAASAASGSPGPGEGSAGGEKR q value: 8.7291e−05 Tr_novel:TRUE RefSeq_Novel:TRUE
Search result spec prec mz: 701.0032 Actual spec prec mz: 701.00317
Fragments matched per AA: 2.16 Proportion of top 20 peaks matched: 0.3
Intensity
6e+05
4e+05
2e+05
0e+00
500
1000
M/Z
y13
y12
y14*
y15
y16
B
Scatterplot of predicted elution time
Fitting R2: 0.816
Novel peptide residual Z score: 0.633
Number of peptides: 1267
Pred. hydrophobicity
50
25
0
−25
0
20000
40000
60000
Experimental scan number
C
Distributions of residuals from best−fit line
of predicted RT vs Expt. scan number
Line: Z score of novel peptide
Z: 0.633
count
300
200
100
0
−10
−5
0
5
10
Z scores of residuals from best linear fit
